# Disorders in Brain Development and Nervous System: Key Molecules and Pathology

**DOI:** 10.3390/ijms252010901

**Published:** 2024-10-10

**Authors:** Kazuhiko Nakadate, Kiyoharu Kawakami

**Affiliations:** Department of Functional Morphology, Meiji Pharmaceutical University, 2-522-1 Noshio, Kiyose, Tokyo 204-8588, Japan; k-kawakami@my-pharm.ac.jp

Brain development is an extremely complex and essential biological process that begins at the start of life and continues throughout an individual’s lifespan. It is centered on the generation of neurons and the establishment of their function. The brain is composed primarily of neurons and glial cells. Neurons are nerve cells that transmit information via electrical signals, whereas glial cells support and protect neurons and regulate their metabolism. The interaction between these two cell types not only maintains the higher functions of the brain but also facilitates its growth and development. This process is common in many animals, including humans, and evolves as the individuals mature.

Brain development is a biological process of neuron generation, encompassing various key stages including cell proliferation, differentiation, migration, axon guidance, synapse formation, and the establishment of neural networks. The early postnatal period is crucial for long-term health due to the rapid growth of the brain before and after birth. For humans, the first 8 years are particularly significant, as they lay the foundation for future learning, health, and success. However, the brain development continues into adulthood. Both prenatal and postnatal neurons react to various chemical signals that are vital for the development, normal functioning, and impairment of neurons and glial cells within the nervous system. Brain development is influenced by genetic factors as well as environmental elements such as nutrients, toxins, and infections. Compared to the brains of other animals, the human brain is more complex and undergoes rapid development after birth. An infant’s brain nearly doubles in mass within the first 6 months, reaching 80% of the adult brain size by the age of 2 years [1]. By contrast, other mammals have brains that closely resemble adult brains in both size and complexity. Even chimpanzees, our nearest evolutionary cousins, have brains at birth that exceed 40% of the adult brain size [2], whereas the brains in human newborn brains are less than 30% of the adult size. Scientists estimate that for a human infant’s brain to develop similarly to that of a baby chimpanzee’s, gestation would need to last between 18 and 21 months [3]. However, extended pregnancy is not feasible because the infant’s head is too large to pass through the birth canal. Consequently, human babies are born with relatively underdeveloped brains.

**Developmental processes of neurons and their importance**

Brain development proceeds through multiple stages. First, neural tube formation occurs early during embryonic development, which serves as the foundation for the brain and spinal cord. Later, neural stem cells divide to produce neurons and glial cells, which constitute the basic structure of the brain. During the proliferative phase of neuronal development, tens of billions of neurons are generated, which differentiate and acquire specific functions. Differentiated neurons migrate to specific sites within the nervous system, a process known as “neuronal migration,” where they are placed in precise locations. Once neuronal migration is complete, the next process, called axon guidance, is initiated. Axons are long projections that allow neurons to send signals to other neurons or target cells, following paths dictated by chemical signals to reach their precise targets. Axon guidance is a precise process, and errors can lead to information transmission defects. Once the axon reaches the target cell, a synapse is formed. Synapses are connection points where neurons exchange information with each other and where electrical and chemical signals are transmitted. Synaptogenesis is extremely important in brain development, particularly during the early developmental stages after birth. During this period, the brain has a high degree of plasticity and neural circuits change dynamically in response to external stimuli and experiences. Appropriate stimulation and experience promote synapse formation and efficient information transfer. Conversely, improper synapse formation or insufficient stimulation from the environment during this period may lead to incomplete neural circuit development, which may adversely affect future cognitive functions and behaviors.

**Fetal brain development**

Prenatal brain development has a particularly significant impact on postnatal brain development. The basic structure of the brain is formed during the early phase of pregnancy; however, during the mid- to late-phase of pregnancy, the proliferation of neurons progresses rapidly, and neural circuits begin to form. During this period, neuronal development may be inhibited if the mother does not receive adequate nutrition or is exposed to toxins. For example, folic acid deficiency causes neural tube defects, and iron deficiency can adversely affect fetal brain development. Alcohol, tobacco, and drug consumption have been reported to have serious effects on the fetal brain [4,5,6]. Fetal alcohol syndrome is a developmental disorder caused by alcohol consumption during pregnancy, which can lead to intellectual disabilities and behavioral abnormalities [7]. This can result in abnormal neuronal differentiation and migration and the incomplete formation of neural circuits.

**Brain development after birth**

Brain development progresses rapidly after birth, especially during infancy. The first few years of life are a period of active synapse formation, and the environment and experiences provided during this period have a significant impact on brain growth. Rich sensory experiences and social stimulation increase the number of synapses and aids in efficient neural circuit formation. This process “synaptic pruning”, ensures that unnecessary synapses are eliminated, allowing efficient neural circuits to remain. However, if sensory and social stimulation are lacking, brain development may be delayed. Studies have shown that an appropriate attachment relationship of infants with their mother positively influences their brain development. Conversely, children who are abused or neglected have been shown to be more likely to experience negative effects on brain development.

**Influence of the central nervous system and chemical factors**

Brain development is greatly influenced by the chemical environment of the central nervous system. Many chemical factors are involved in the differentiation and development of neurons, and the normal formation of neural circuits occurs when these factors work in balance. Nerve growth factor and brain-derived neurotrophic factor (BDNF) play central roles in neuronal growth and synapse formation. When these molecules do not function properly, brain development can be impaired. In addition, glial cells support neuronal function by supplying nutrients to neurons, processing waste products, and contributing to the formation and maintenance of synapses. Disruptions of these cellular interactions can adversely affect the development and subsequent functioning of neural circuits.

**Brain plasticity and lifelong development**

While brain development progresses rapidly during childhood and adolescence, the brain continues to change constantly in adulthood. Neuronal plasticity persists in adulthood, allowing new experiences and learning to reorganize neural circuits. This plasticity plays an important role in learning and memory processes, indicating that the brain has the capacity to adapt throughout life. It has been reported that biogenic amines (serotonin, acetylcholine, and noradrenaline, etc.) are involved in “synaptic pruning” and may significantly affect brain function later in life [8,9,10]. These biogenic amines are strongly influenced by the autonomic nervous system, even after adulthood, suggesting that they may be deeply involved in brain activity depending on the environment. However, with aging, this plasticity gradually diminishes and the ability of neurons to regenerate becomes limited. Aging typically results in a reduction in the number of synapses and decreases the efficiency of neurotransmission, which can result in cognitive decline and memory loss. Nonetheless, proper lifestyle and stimulation are important for maintaining brain health.

**Lifestyle and its impact on brain health**

In recent years, research has been conducted on the effects of lifestyle changes on brain health. A lack of sleep and chronic stress have been shown to negatively affect the brain’s neurons and neural circuits. Because the brain organizes information and consolidates memories during sleep, a lack of sleep can interfere with these processes, leading to impaired learning and cognitive function. Stress can also affect the hormonal balance in the brain and chronic stress in particular can reduce the number of neurons in the hippocampus [11,12]. This can lead to difficulties with memory and emotional regulation and can increase the risk of depression and anxiety disorders. In addition, a lack of exercise and improper diet are known factors that can negatively impact brain health [13]. For example, obesity and diabetes have been reported to increase the risk of developing cerebrovascular diseases and dementia. However, proper exercise and a balanced diet are critical for maintaining brain health. Exercise promotes BDNF secretion, which supports neuronal growth and synapse formation [14]. In addition, foods rich in omega-3 fatty acids and antioxidants have been shown to reduce brain inflammation and improve cognitive function [15,16].

**Environmental changes and their effects on the brain**

Environmental changes in modern society have also been identified as factors that affect brain development and health. Exposure to air pollution and harmful chemicals can negatively affect brain development [17]. During fetal development and early childhood especially, these environmental factors can have a direct impact on the formation of neural circuits. In addition, concerns have been raised regarding the excessive use of electronic devices and prolonged screen time, which can negatively impact brain development. Studies have shown that increased screen time negatively affects attention, concentration, and social skills, particularly in children. This may delay brain development, particularly affecting the development of the prefrontal cortex. The prefrontal cortex is involved in judgment, decision-making, and emotion regulation; therefore, the impact of increased screen time in this area is serious.

**Conclusion**

Brain development is a complex process that begins before birth and continues throughout the life. This process includes the generation, differentiation, and migration of neurons, axon guidance, synapse formation, and formation of neural networks. These processes are significantly influenced by environmental, chemical, lifestyle, and genetic factors. Proper nutrition, stimulation, and healthy lifestyle habits during fetal development and early childhood are crucial for brain development, which, in turn, significantly impacts future learning abilities and health.

The identification of the “key molecules” that function in vivo is important to understand brain development and diseases and to establish therapeutic and preventive measures. Various proteins and molecules play important roles in neuronal differentiation, synapse formation, and the development of neural circuits. Elucidating the specific functions of these molecules and their regulation of neural circuits will significantly contribute to our understanding of brain function. Therapeutic strategies targeting the key molecules may lead to the development of novel treatments for neurological diseases. Furthermore, histopathological analysis is essential for understanding brain function. This analysis enables the direct observation of the morphological changes in neurons and glial cells, synaptic conditions, and abnormalities in neural circuits to understand these changes at the cellular level. Studying abnormalities in brain function, such as developmental disorders and neurodegenerative diseases, is particularly valuable. By clarifying the molecular and cellular mechanisms underlying brain dysfunction, histopathological analysis will greatly contribute to the development of therapeutic and preventive measures.

Therefore, the identification of the key molecules that function in vivo, in combination with histopathological analysis, is critical for maintaining brain health, understanding brain function, and treating brain diseases. This will help to deepen our understanding of brain development and function, the pathophysiology of disease, and provide new insights into future medicine.
